# Universality of Schmidt decomposition and particle identity

**DOI:** 10.1038/srep44675

**Published:** 2017-03-23

**Authors:** Stefania Sciara, Rosario Lo Franco, Giuseppe Compagno

**Affiliations:** 1 INRS-EMT, 1650 Boulevard Lionel-Boulet, Varennes, Québec J3X 1S2, Canada; 2 Dipartimento di Fisica e Chimica, Università di Palermo, via Archirafi 36, 90123 Palermo, Italy; 3 Dipartimento di Energia, Ingegneria dell’Informazione e Modelli Matematici, Università di Palermo, Viale delle Scienze, Edificio 9, 90128 Palermo, Italy

## Abstract

Schmidt decomposition is a widely employed tool of quantum theory which plays a key role for distinguishable particles in scenarios such as entanglement characterization, theory of measurement and state purification. Yet, its formulation for identical particles remains controversial, jeopardizing its application to analyze general many-body quantum systems. Here we prove, using a newly developed approach, a universal Schmidt decomposition which allows faithful quantification of the physical entanglement due to the identity of particles. We find that it is affected by single-particle measurement localization and state overlap. We study paradigmatic two-particle systems where identical qubits and qutrits are located in the same place or in separated places. For the case of two qutrits in the same place, we show that their entanglement behavior, whose physical interpretation is given, differs from that obtained before by different methods. Our results are generalizable to multiparticle systems and open the way for further developments in quantum information processing exploiting particle identity as a resource.

Systems of identical particles constitute the basic building blocks of quantum information theory, being present in Bose-Einstein condensates^1^,^2^, quantum dots^3^,^4^,^5^,^6^, superconducting circuits^7^ and optical setups^8^,^9^. Completely characterizing the quantum features of these composite systems is thus a crucial requirement from both fundamental and technological viewpoint. Investigation of bipartite entanglement for identical particles started some time ago^10^,^11^,^12^,^13^,^14^,^15^ but, differently from the case of distinguishable particles, the subject has remained controversial^10^,^11^,^12^,^13^,^14^,^15^,^16^,^17^,^18^,^19^,^20^,^21^,^22^,^23^,^24^,^25^,^26^,^27^. The controversy mainly arises from the way identical particles are ordinarily treated in quantum mechanics by the standard particle-based approach, that is by making them artificially distinguishable with the attribution of nonobservable labels^27^,^28^. This practice has the consequence that the structure of the states inevitably is, with respect to labels, that of an entangled state. As a consequence, some viewpoints has been advanced^12^,^14^,^16^,^17^,^18^,^19^,^20^,^21^,^22^,^23^,^24^,^25^ differing both in interpretation and, in some cases, in the quantification of the part of entanglement attributable to particle identity. The last aspect can hardly be considered devoid of importance in view of entanglement being in general a resource for quantum information and communication^12^,^14^,^18^,^29^,^30^,^31^,^32^,^33^,^34^,^35^,^36^,^37^. Differences among the viewpoints clearly emerge in the simple paradigmatic case of identical particles independently prepared in far regions, such that they are expected not to have any correlations, and then allowed to merge spatially without any other change. In one viewpoint, as epitomized in a textbook^27^, the entanglement due to indistinguishability is present for far particles but nevertheless is physically no matter of concern because it cannot be exploited. In a widely held second viewpoint^18^,^26^, this entanglement is a merely formal artifact even when the particles are brought to overlap. In order to characterize it in the latter situation, a third viewpoint has been proposed which resorts to extraction procedures^17^. The consequent conclusions may be however subject to criticism on the ground that the extracted entanglement does not represent the one in the overlapping condition being instead produced by the extraction operation itself.

Among tools which are at the heart of quantum information and quantum computation, there is the Schmidt decomposition (SD) for bipartite systems of multilevel particles in pure states. It is of general application to entanglement characterization, theory of measurement, state purification, quantum erasure^29^,^30^ and also in black-hole physics^38^,^39^. Despite its wide utilization in systems of distinguishable particles, even the SD remains debated for identical particles^10^,^16^,^26^ where it is replaced by Slater decomposition. The associated Slater rank witnesses entanglement but its interpretation is different for bosons or fermions^26^. For distinguishable particles, the SD unveils the entanglement of the system by the von Neumann entropy of the reduced density matrix, whose eigenvalues are the squares of the Schmidt coefficients appearing in the decomposition^30^. Instead “the relationship between Schmidt coefficients and the eigenvalues of the reduced density matrix breaks down in the case of identical particles”^26^. Therefore, the ordinary notion of partial trace to get the reduced state has not been considered suitable for assessing the entanglement of identical particles^16^,^18^,^26^. Attempts to provide a SD for indistinguishable particles give wrong results, as nonzero entropy and thus the presence of entanglement for uncorrelated fermions^10^.

Using a recent non-standard particle-based approach^40^, here we develop a SD valid for both bosons and fermions, which allows the direct characterization of the physical entanglement of the particles. Differently from previous viewpoints, application of this method to the paradigmatic example exposed above allows, without resorting to extraction, to demonstrate that entanglement is zero for separated particles and nonzero when they overlap. Moreover, our SD overcomes the problems occurring in other proposals of SD within the second quantization^10^, being the Schmidt coefficients always the square roots of the eigenvalues of the reduced density matrix, exactly as for distinguishable particles. This SD gives the novel tool to straightforwardly characterize the entanglement for identical particles.

## Results

### Theory

We recall the notation of the intrinsically symmetric particle-based approach introduced in ref. 40. Hereafter, we mean by “symmetric states” (or “symmetric Hilbert space”) the symmetric or antisymmetric behavior of the system states depending on the bosonic or fermionic nature of the particles, respectively. The overall state of two identical particles, one in the state *ϕ* and one in *ψ*, is completely characterized by enumerating the one-particle states and represented as |*ϕ, ψ*〉. Two particles in |*ϕ, ψ*〉 are not independent and their overall state is a whole which cannot be written as a tensorial product of one-particle states, i.e. 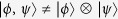. However, a nonseparable external symmetric product of one-particle states (wedge product) can be introduced as 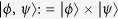. Analogously, we have 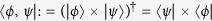 (this wedge product will be crucial in demonstrating the theorem below). The probability amplitude of finding the two particles in 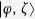 if they are in |*ϕ, ψ*〉, is obtained by the symmetric two-particle scalar product defined in terms of one-particle amplitudes as^40^


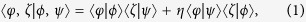


where *η* is +1 for bosons and −1 for fermions. This probability amplitude immediately shows that the generic state |*ϕ, ψ*〉 is symmetric, i.e. 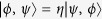. The state |*ϕ, ψ*〉 spans a linear symmetric two-particle Hilbert space 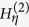. A symmetric inner product between state spaces of different dimensionality (one-particle projective measurement) can also be introduced as^40^


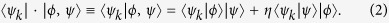


In 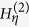 it is possible to choose an orthonormal two-particle basis 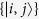, |*i*〉 and |*j*〉 being single-particle states, where an arbitrary state of two identical particles can be expressed as 
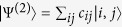
. By Eq. (2), one then gets the reduced (single-particle) density matrix via partial trace as^40^ 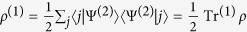, where 
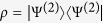
. We emphasize that now partial trace depends on the single-particle basis being local or nonlocal, as we shall show in the following. This behavior differs from the case of distinguishable particles where, since a single-particle basis always addresses a given particle, the partial trace is not affected by the local or nonlocal nature of the basis. We can now give the following theorem.

### Theorem 1

Within a symmetric two-particle Hilbert space 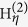, a pure state of two d-level identical particles |Ψ〉 can always be written in the Schmidt decomposition (SD)


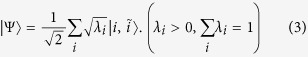


The “Schmidt coefficients” 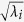 are the square roots of the eigenvalues of the reduced density matrix and the states 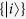 its eigenstates. The state 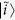 belongs to the basis 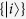 and the symmetric two-particle basis 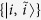 is the “Schmidt basis”.

*Proof*. We express the state |Ψ〉 in terms of the symmetric two-particle basis 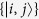 as 
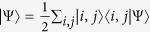
, where the symmetric two-particle identity matrix 
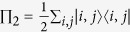
 has been inserted. By defining 
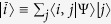
, the state can be further cast as 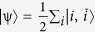. Generally, the states 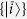 are not orthonormal. Nevertheless, as for distinguishable particles^29^, there exists a basis 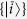 where they are orthogonal, i.e. 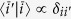. We thus write


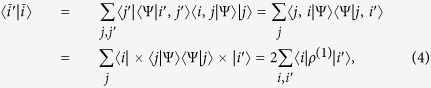


where we have used the partial trace 
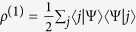
. When the states 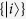 are the eigenstates of 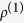, i.e. 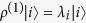, the states 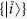 are orthogonal and satisfy 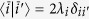. Denoting by 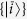 the set of orthonormal states associated to 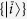, we have


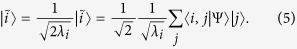


Both 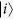 and 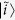 are eigenstates of *ρ*^(1)^ with the same eigenvalue *λ*_*i*_ (as proven in Methods). Thus, given a set of eigenstates 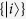, each 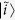 is one of the states within the set. For bosons, if the eigenvalues are nondegenerate then 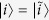; for fermions, Pauli exclusion principle dictates 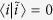 and the eigenvalues are always degenerate. Substituting 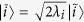 of Eq. (5) in 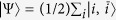, the SD of Eq. (3) is finally demonstrated. ◽

When the states are characterized by more than one observable, for instance when the single-particle basis is 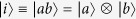 (|*a*〉 and |*b*〉 being two independent observables), one can be interested in studying the system for a fixed value of one of the observables. In such cases, the theorem above needs to be specialized. Let us take a two-particle state of the form 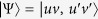, where *u, v* are arbitrary single-particle states. This means that the SD of 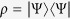 is obtained by following the theorem above with the difference that the partial trace is now performed on the subspace of *b (a*) with the observable *a (b*) fixed. The corresponding reduced density matrix is indicated as 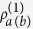 (see Methods). The universality of SD just proven entails its application to identical particles in many scenarios of quantum information (entanglement characterization, purification, measurement theory) in analogy with distinguishable particles^30^. Knowledge of the Schmidt basis is essential to find the suitable set of measurements (Schmidt observables^41^) to acquire information on correlated identical particles in experimental contexts^42^,^43^,^44^.

The SD of Eq. (3) defines an entangled state in terms of nonseparability, whatever the overlap between the particles. As for distinguishable particles^29^, we define the positive integer “Schmidt number” *s* as the number of terms appearing in Eq. (3), that is the number of nonzero eigenvalues of 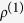. If *s* = 1, 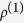 is pure and identifies a nonentangled state; if s >1, 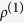 testifies an entangled state. The Schmidt number thus acts as entanglement witness. Analogous considerations hold for 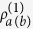. In particular, the (symmetric) basis state 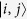 with single-particle states |*i*〉, |*j*〉 containing only one observable results to be unentangled when 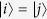, while it is maximally entangled when 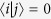 (see Methods). Being the Schmidt coefficients 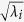 the square roots of the eigenvalues of the single-particle reduced state, they immediately lead to the von Neumann entropy


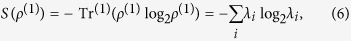


as a quantifier of entanglement for identical particles, exactly as happens for nonidentical particles^29^. Given any pure state 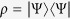, its SD is obtained in a recipe format as follows:

- perform the trace of *ρ* on a chosen single-particle basis to get the reduced single-particle density matrix 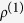 (or 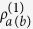);
- calculate eigenvalues, *λ*_*i*_, and eigenstates, 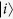, of 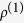 (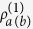);
- construct the states 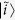 and express the state 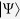 in terms of the Schmidt basis 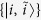.

### Applications

**Figure 1:**
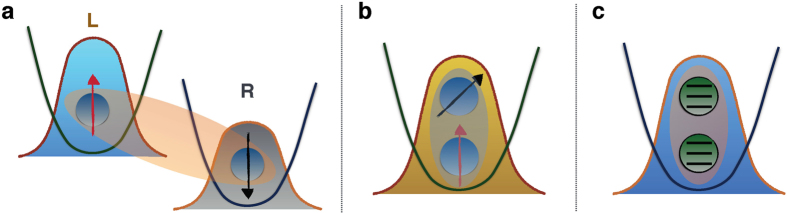
(**a**) Two identical qubits in two spatially separated places with opposite pseudospins. (**b**) Two identical qubits in the same spatial mode with arbitrary pseudospins. (**c**) Two identical qutrits (three-level quantum systems) in the same spatial mode. The shaded ellipses indicate that the particles are entangled.

In the following, we apply this recipe to some states of interest (see Fig. 1). The first one is a situation already known^40^ which is here particularly useful to present how our method works. The other ones are new examples which evidence the usefulness of SD in finding novel entanglement features of identical particles.

#### Two qubits in two separated sites (Bell-like state).

 We consider two identical particles (bosons or fermions) with orthogonal internal degrees of freedom (pseudospins) located in separated sites, described by


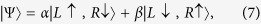


where 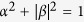 (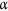 real, 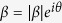 with *θ* being the relative phase). The site *M (Left (L*) or *Right (R*)) and the pseudospin *σ* (↑, ↓) are independent observables. The two sites are nonoverlapping, behaving thus as “physical” labels. The state of Eq. (7) recalls Bell-like states^26^,^45^. It permits us to discuss the role of local and nonlocal measurement in the structure of the SD for identical particle states. When a single-particle property (e.g., the pseudospin) is measured in a localized region of space (e.g., *L*), the measurements itself and the corresponding partial trace are *local*^40^. According to the recipe above, this measurement corresponds to project 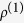 on the local basis, i.e. on the subspace 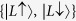. The reduced single-particle density matrix is


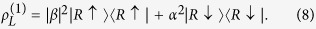


It has eigenvalues 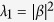, 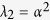, and eigenstates 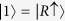, 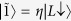, 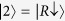, 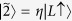, which define the Schmidt basis. We notice that 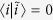 and that the particle statistics is intrinsically included by the presence of 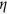. The SD of 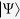 is


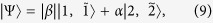


with von Neumann entropy


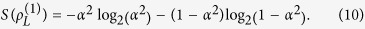


This result coincides with the known von Neumann entropy for two distinguishable particles in a Bell-like state, giving maximal entanglement 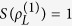 for a Bell state (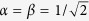)^31^.

When nonlocal (one-particle) measurements are performed simultaneously on both sites (*L* and *R*) where the particle has nonzero probability of being found, the trace is *nonlocal*. Operationally, it corresponds to perform the partial trace of *ρ* on the global single-particle basis, i.e. on 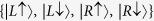. Following the recipe above by a global partial trace, we get a SD of the Bell-like state 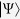 different from Eq. (9) (see Methods) leading to


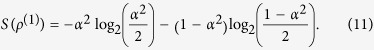


The difference between 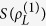 and 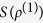 highlights the importance of measurement localization on the structure of the SD and in turn on the entanglement between two identical particles located in different sites. To further clarify this aspect, we consider the particular case α = 1 when the state 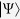 becomes 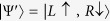, which is unentangled^40^ since the particles are in separated sites and behave as uncorrelated distinguishable particles^31^. For this state, 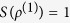 and 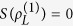. For systems of identical particles, local single-particle measurements supply the intrinsic entanglement^40^, whilst nonlocal measurements yield “measurement-induced entanglement”^26^,^46^. This feature must be contrasted with what happens for distinguishable particles, where single-particle measurements always address individual particles. We point out that these results overcome the issue existing with previous proposals of Schmidt decomposition and entanglement measure for identical particles which give nonzero entanglement for uncorrelated separated particles^10^,^11^.

#### Two qubits in the same site with arbitrary pseudospins

**Figure 2:**
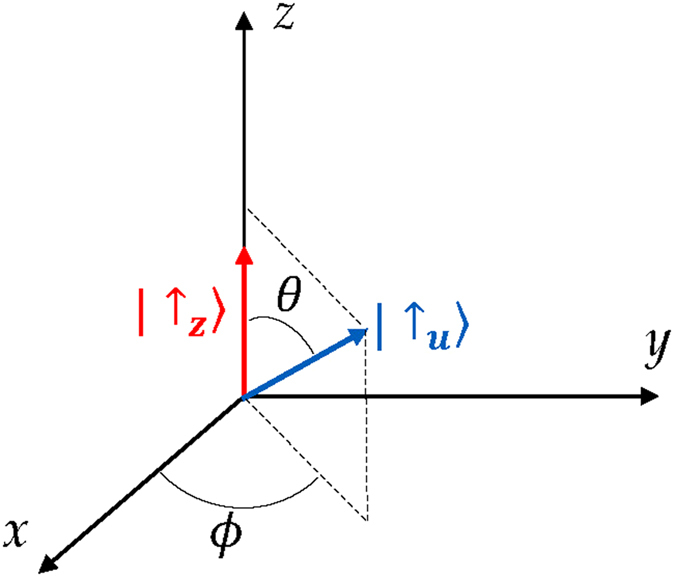
The two-qubit state is expressed by 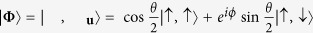 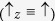. One spin (red arrow) is along 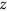-direction and the other (blue arrow) in the direction determined by the angles 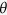 and 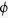.

**Figure 3:**
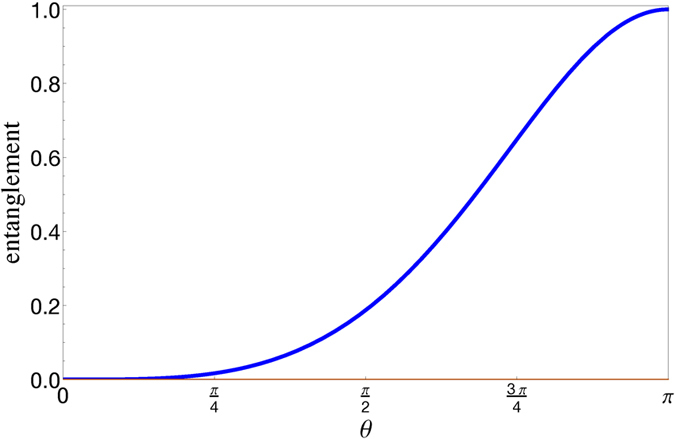
Entanglement quantified by the von Neumann entropy of the state 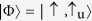, 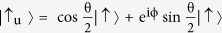 is plotted as a function of ***θ***.

Entanglement is a measure of nonseparability of the state^47^. When the particles are in the same site, their internal states (pseudospins) establish such nonseparability. A recent experiment showed that the entanglement in a Cooper pair can be extracted by means of graphene quantum dots, so that it can be possibly used as a resource for quantum information in the solid state^4^. Moreover, it was recently observed^48^ that it is possible to prepare two maximally entangled ultra-cold atoms with opposite spin states by bringing them into the same optical tweezer (site). In general, one physically expects that situations may occur where particles are in the same site *M* with pseudospins in arbitrary directions. Such a condition is possible only for bosons, since for fermions the only allowed state by the Pauli exclusion principle is that with opposite pseudospins which is maximally entangled^40^. We hence study two identical boson qubits (e.g., photons) with one pseudospin along *z*-direction (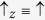) and the other one along the direction 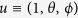, as displayed in Fig. 2 (this situation generalizes that of two bosons with opposite pseudospins treated previously^40^). By exploiting linearity and omitting the spatial index *M*, this state has the (unnormalized) form


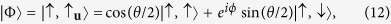


where 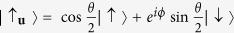. Following the recipe above by performing the partial trace on the basis 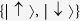 (see Methods), we obtain its (normalized) SD


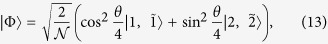


where 
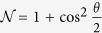
, 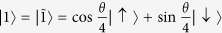 and 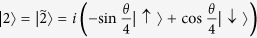. Notice the dependence of SD on *θ*, which represents the pseudospin state overlap of the two particles. Entanglement of the two boson qubits is quantified by the von Neumann entropy 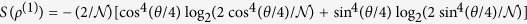 and plotted in Fig. 3. It is maximum for *θ* = *π* (opposite pseudospins) and zero for *θ* = 0 (same pseudospins).

Two indentical qubits in the same site can thus be physically entangled^40^, a result which is indirectly confirmed by extraction procedures^17^ and is instead uncaught by other entanglement measures for identical particles, like the detection-level concurrence^26^.

#### Two identical qutrits in the same site

Systems of three-level particles (also called qutrits) are promising alternative candidates to be used in quantum processors instead of the standard two-level qubits^49^,^50^. We apply our method to two identical qutrits in the same site, each characterized by the basis 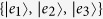 (the spatial index is omitted for simplicity). Specifically we take this system, which is equivalent to that of two spin-1 bosons, in the previously analyzed state^16^.


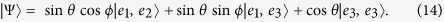


By the partial trace on the above one-particle basis, we obtain the following reduced density matrix


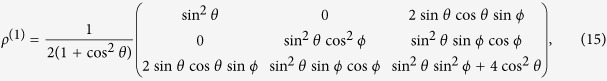


from which one can calculate the eigenvalues and eigenvectors required for constructing the SD. Since their explicit expressions are cumbersome, we focus on a simple particular case which allows us to make the comparison to another method^16^ that provides different entanglement predictions for this state. In particular, we choose 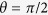 in the state of Eq. (14), which thus reduces to


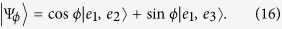


**Figure 4:**
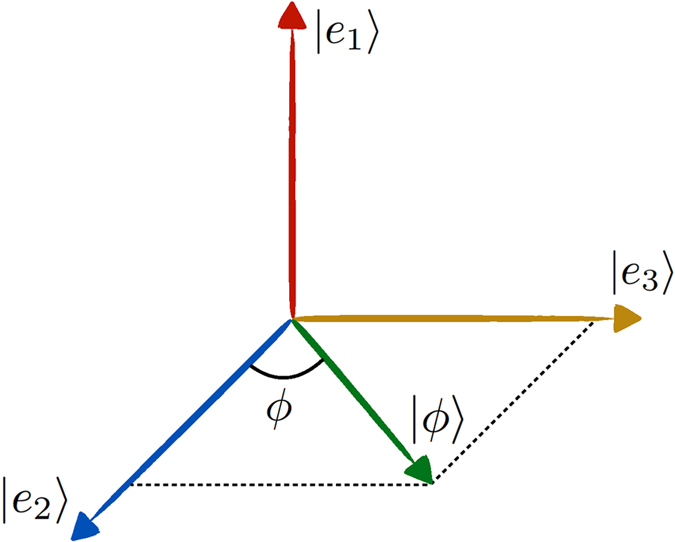
The two-qutrit state is expressed by 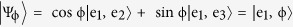, where 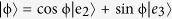. The single-particle states 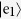 and 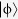 are orthogonal.

By following the usual recipe (see Methods), we get its SD


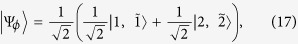


where 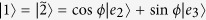, 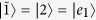, 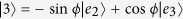, 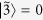. Expressing 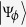 in the single-particle basis 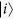 (*i* = 1, 2, 3) and exploiting the linearity of the symmetric Hilbert space^40^, we get 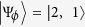. The von Neumann entropy of Eq. (6) is 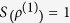, which represents a maximally entangled state independently of *ϕ*. We provide a physical motivation to support this result. We notice that 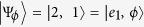, where 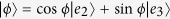. The independence of *ϕ* is due to the fact that the amount of entanglement only rests on the scalar product and hence on the angle between the single-particle states 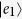, 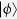, as depicted in Fig. 4. Moreover, entanglement is maximum because 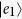, 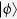 are orthogonal (as mentioned in *Theory* section and demonstrated in the Methods). This situation is analogous to the case of two identical qubits in the same site with pseudospin states in arbitrary directions 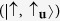 treated before. Our result (*ϕ*-independent) contrasts with the previous one (*ϕ*-dependent)^16^.

## Discussion

We observe that a difference exists from an operational viewpoint between nonidentical and identical particles. For distinguishable particles, SD and its corresponding entanglement are known to be exploitable within a resource theory by local operations, addressing each individual particle, and classical communication (LOCC)^29^,^30^. Differently, indistinguishable particles are not individually addressable. Nevertheless, the SD here presented for identical particles identifies an entanglement still exploitable by LOCC, which then allows quantum information protocols like teleportation. As an instance, this can be achieved by resorting to extraction procedures which make the overlapping identical particles tunnel with certain probabilities into two separated spatial modes^17^. For particles in the same site, where an intrinsic entanglement can be defined^40^, it is straightforward to realize that the original Schmidt decomposition is reproduced, in a conditional fashion, into the two-particle state of the two accessible separated modes. The operational aspects will be treated elsewhere in detail, including the case of partially overlapping identical particles where the definition of entanglement is more subtle^40^.

Summarizing, we have supplied, within a non-standard approach to identical particles^40^, a universal SD of bipartite quantum systems, holding for particles of any nature. This result shows that the problems arising with other attempts of SD for identical particles within the second quantization, which are solved here in a natural way, are not therefore to be settled by arguments related to quantum information coding and processing^10^. In our approach the Schmidt number maintains its role of entanglement witness and the Schmidt coefficients can be faithfully used to calculate the von Neumann entropy. These aspects permit, differently from what has been claimed^12^,^26^,^31^, unambiguous quantification of entanglement for indistinguishable particles by ordinary notions, like the von Neumann entropy after partial trace.

We have first tested the reliability of the SD by using it to study the well-known condition of two identical qubits with opposite pseudospins in spatially separated sites. The SD and the corresponding entanglement entropy give the physically expected results, as zero entanglement for a product state and maximal entanglement for a Bell state, also showing that nonlocal measurements induce entanglement in the system. We have then applied the SD to analyze two boson qubits in the same site, finding how the amount of their entanglement depends on their pseudospin overlap: the entanglement increases as the two internal states tend to be orthogonal. This behavior generalizes previous results limited to orthogonal pseudospins^40^, which appear to confirm recent experimental observations of entanglement extraction in Cooper pairs^4^ and of entanglement generation between two cold atoms in the same optical tweezer^48^. We have finally studied a system of two identical qutrits, which are relevant for storing quantum information^49^,^50^. We have straightforwardly obtained their entanglement and provided a physical interpretation in the case when they are in the same site. Our result differs from that determined for the same system by an alternative approach^16^. The origin of this difference in the entanglement measurement remains to be understood, requiring experimental verification and comparison of both theoretical approaches.

Our result allows the natural generalization of the SD to arbitrary bipartitions of systems of 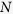 identical particles. Our work enables the exploitation of this tool for characterizing composite quantum systems in theoretical and experimental relevant conditions where identical particles live in partially overlapping sites (e.g., electrons in quantum dots^3^,^4^,^5^,^6^, Bose-Einstein condensates^1^, solid-state qubits in circuit quantum electrodynamics^7^ and wave-guided and integrated photons^8^,^9^), which remain little explored. Our research demonstrates that, differently from what stated before, two identical particles prepared independently in spatially separated sites are not entangled and that, when these particles are brought to overlap with no other change, there is a physical entanglement which is to be attributed to quantum indistinguishability. This fact settles the ambiguity on the interpretation of identical particle entanglement and establishes that entanglement between identical particles is not a mere mathematical artefact, as has been argued^26^,^18^. As a further novel aspect, our approach evidences how the local and nonlocal nature of single-particle measurements, which define the partial trace operation, and the single-particle state overlap influence the structure of the SD and therefore the quantification of the entanglement. The Schmidt decomposition for identical particles here reported supply methods to exploit the resources of entanglement coming from particle identity for applications such as state teleportation, quantum metrology and quantum cryptography.

## Methods

### Eigenstates 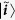 of the reduced density matrix

Here we demonstrate that the states 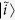 are eigenstates of 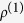 with eigenvalues 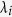, analogously to the eigenstates 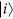. We start by using Eq. (5) of the manuscript and reminding that 
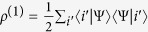
 to have


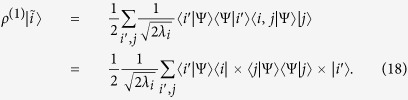


Since 
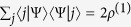
 and 
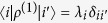
, we obtain


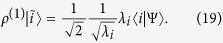


At this point, inserting the two-particle identity matrix 
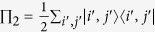
 between 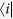 and 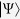 and using Eq. (2) of the main text to get 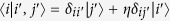, we find


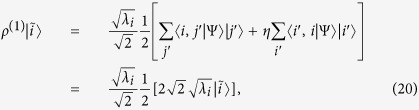


where the last equality is due to Eq. (5) of the manuscript and to the symmetry property 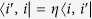 (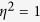). Hence, we conclude that


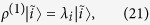


that is what we intended to demonstrate. Notice that the states 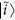 belong to the basis 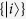 of the eigenstates of the reduced density matrix.

### Relationship between the eigenstates 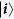 and 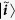


According to Eq. (5) of the manuscript, one has


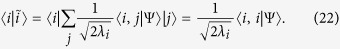


Expressing 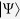 by the SD of Eq. (3) of the main text, we obtain


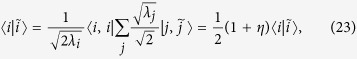


where we have used 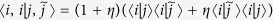 (see Equation (1) of the manuscript) and 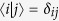.

From the previous equation, it is immediately seen that for fermions, as expected, it is always 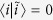, since two of them cannot occupy the same state (Pauli exclusion principle). The orthogonality of the eigenstates 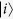 and 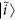 implies that the eigenvalues 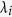 of the reduced density matrix for a state of two fermions must be degenerate. For states of two bosons, instead, both cases of degenerate and non-degenerate eigenvalues can occur. In particular, if the eigenvalues 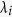 of the reduced density matrix are non-degenerate, it immediately follows 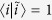: the eigenstates 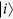 and 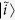 coincide. We stress that these properties are always true when the eigenvalues of the reduced density matrix are calculated within the complete single-particle basis (including all possible outcomes of the observables which define a single-particle state) or in the specific case when the single-particle state is described by an observable alone, which are the conditions assumed in proving the theorem of the manuscript. Wider scenarios arise when the reduced density matrix is instead calculated by fixing a given value of an observable.

### Partial trace on a given subspace of an observable

Let us consider a single-particle state 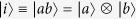 and a two-particle state 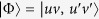, where 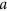, 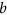, 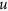, 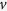, 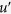, 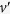 are arbitrary states corresponding to two independent observables 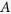 and 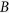 (e.g., the site and the spin of the particle). We show a general criterion to perform the partial trace of 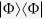 on the subspace of an observable (e.g., 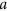) by varying the other one (e.g., 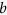). We first calculate the one-particle projective measurement (see Equation (2) of the manuscript)


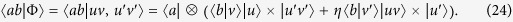


The action of 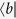 on the state 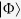 can be thus defined as


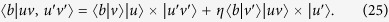


The reduced single-particle density matrix performed on the subspace 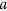 of the observable 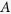 (that is, obtained by fixing 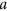 and summing on 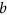) reads


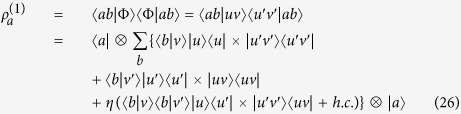


Once the reduced density matrix is so obtained and normalized, the entanglement can be quantified by von Neumann entropy, as usual.

### Entanglement of a two-particle basis state 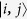


Here we calculate the entanglement of a basis state 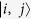 within the complete single-particle basis, showing that it depends on the scalar product between 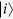, 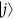. We thus consider the two-identical particle state 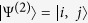, where 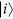 and 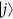 are generic single-particle states. The reduced single-particle density matrix, performed on the basis 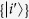, reads


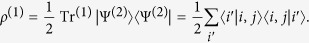


By using Eq. (2) of the manuscript, we obtain


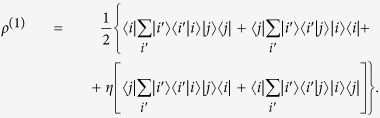


Recognizing the presence of the single-particle identity 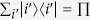, the reduced density matrix 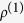 reduces to


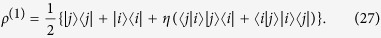


If 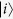 and 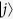 are orthogonal, i.e. 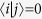, one obtains


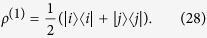


Since the eigenvalues of 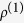 are 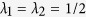, entanglement as quantified by the von Neumann entropy is maximum (see Equation (6) of the main text). Moreover, the presence of entanglement (independently of its amount) is witnessed by the number of the nonvanishing eigenvalues, that is by the Schmidt number.

On the other side, when the states 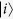, 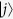 coincide, that is 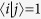, one obtains


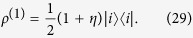


Such a condition, allowed only for bosons (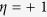), leads to 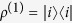 which is a pure state whose unique nonvanishing eigenvalue is 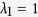. Entanglement of this state is thus zero (the von Neumann entropy vanishes), as already witnessed by the presence of only one nonvanishing eigenvalue in the reduced density matrix.

We then conclude that the entanglement of a two-particle basis state 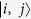 depends on the scalar product (and thus on the angle, from a geometrical viewpoint) between the single-particle states 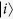 and 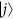. It is maximum when they are orthogonal and zero when they are the same. Furthermore, we have here confirmed that the Schmidt number is an entanglement witness, as for distinguishable particles^29^. We remark that these results are valid when the partial trace is performed within the complete single-particle basis in the specific case when the single-particle state is described by an observable alone. We have already seen (see section above and the case of two spatially separated particles of the manuscript) that for a single-particle state described by a number of observables, new scenarios surface for determining the entanglement of the identical particle system.

### Schmidt decomposition of the Bell-like state by a global partial trace

We give here the Schmidt decomposition of the Bell-like state 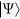 of Eq. (7) of the manuscript, by following the recipe in the main text. We first perform the global partial trace of 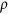 on the total single-particle space 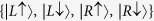, and obtain


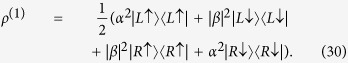


It has eigenvalues 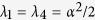, 
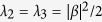
, and eigenstates 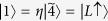, 
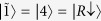
, 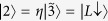, 
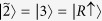
, which define the Schmidt basis. The Schmidt decomposition of the Bell-like state thus results


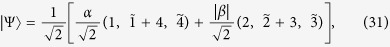


and it permits to write the von Neumann entropy 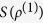 of Eq. (11) of the manuscript. Notice the difference between the SD given here and that reported in Eq. (9) of the main text.

### Schmidt decomposition of the two-boson state |Φ〉

We here provide the Schmidt decomposition of the two-boson state 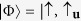 defined in Eq. (12) of the manuscript. The reduced density matrix 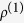, obtained by performing the partial trace of 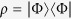 on the basis 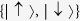, is


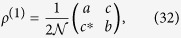


where 
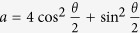
, 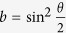, 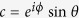 and 
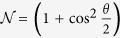
. It is straightforward to find its eigenvalues


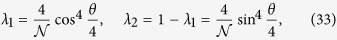


and the corresponding eigenstates


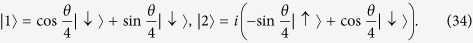


As we see, they only depend on the angle between the pseudospins (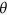). Since we are dealing with two bosons in the same site, whose single-particle states are described by only an observable (the pseudospin), and the eigenvalues are nondegenerate, the single-particle states 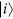, 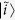 defining the Schmidt basis 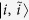 are 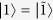 and 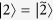. Therefore, the (normalized) Schmidt decomposition of the state 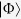, obtained by Eq. (3) of the manuscript, is given by


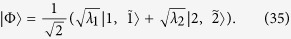


The corresponding entanglement is quantified by the von Neumann entropy 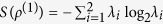.

### Schmidt decomposition of the state |Ψ_*ϕ*_〉 of two qutrits in the same site

We give the Schmidt decomposition of two identical qutrits in the same site, each characterized by the basis 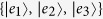. This system is equivalent to that of two spin-1 bosons in the same hole, previously analyzed by an alternative method^16^. We consider the state


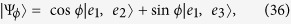


where the spatial index has been omitted for simplicity. By performing the partial trace of *ρ* onto the basis 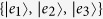, we obtain the reduced density matrix


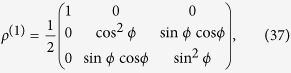


which has eigenvalues 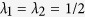, 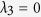 and eigenstates 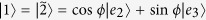, 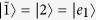, 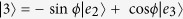, 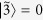, which define the Schmidt basis. From Eq. (3) of the main text, the SD of the state is


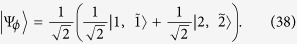


## Additional Information

**How to cite this article**: Sciara, S. *et al*. Universality of Schmidt decomposition and particle identity. *Sci. Rep.* **7**, 44675; doi: 10.1038/srep44675 (2017).

**Publisher's note:** Springer Nature remains neutral with regard to jurisdictional claims in published maps and institutional affiliations.
